# Treatment Response in Pediatric Patients with Status Epilepticus: A Retrospective Observational Study from Saudi Arabia

**DOI:** 10.3390/jcm14175940

**Published:** 2025-08-22

**Authors:** Omar A. Almohammed, Aseel Alsuwayegh, Bader M. Alhadhrami, Abdulaziz A. Alqarni, Marwan A. Alrasheed, Sultan M. Alghadeer

**Affiliations:** 1 Department of Clinical Pharmacy, College of Pharmacy, King Saud University, Riyadh 12372, Saudi Arabia; 2 Pharmacoeconomics Research Unit, College of Pharmacy, King Saud University, Riyadh 12372, Saudi Arabia; 3 Pharmaceutical Care Services, King Saud University Medical City, Riyadh 12372, Saudi Arabia

**Keywords:** status epilepticus, pharmacotherapy, benzodiazepines, anti-seizure medications, response, Saudi Arabia

## Abstract

**Objective:** Investigate patient characteristics, treatments used, treatment response, and factors associated with outcomes when managing SE in a pediatric population admitted to the emergency department (ED). **Methods:** This retrospective observational study included pediatric patients (age ≤ 18 years) with SE admitted to the ED at King Khalid University Hospital between 2015 and 2023. SE and refractory SE (RSE) were diagnosed according to the American Epilepsy Society (AES) definitions. The data included demographics, home medications, treatment sequences, medication dosing, and clinical outcomes. To assess appropriateness, the administered doses were compared with the AES standards for pediatric patients. **Results:** The study included 487 episodes of SE. The mean patient age was 6.1 ± 4.1 years, and most patients were males (57.3%) with a history of epilepsy (74.1%). Benzodiazepines (BDZs) were administered first in 83.0% of cases, with a 10.9% success rate, whereas anti-seizure medications (ASMs) were administered first in 17.0% of cases, with a 66.3% success rate (*p* < 0.0001). Surprisingly, medications administered at appropriate doses during the first round were significantly less effective compared to those that were underdosed (18.2% vs. 28.4%; *p* = 0.0222), mainly because of poor response to BDZs. Younger patients and those who received BDZs on their first medication round had higher hospital admission rates. **Conclusions:** ASMs were more effective than BDZs in managing pediatric patients with SE, regardless of the dosing precision. These findings point toward the adoption of personalized treatment strategies and may warrant early initiation of ASMs. National multicenter studies are needed to define a standardized pediatric SE protocol.

## 1. Introduction

Status epilepticus (SE) is a pediatric neurological emergency characterized by seizures lasting ≥5 min without regaining consciousness or recurrent seizures (≥2) without an intervening period of neurological recovery [1]. SE results from impaired seizure termination or excessive excitatory activity and requires urgent intervention to prevent neurological damage or mortality [2]. SE is more prevalent in children, with 144 cases per 100,000 person-years in infants (1–12 months) and 58 cases per 100,000 person-years in children aged up to 5 years. In Saudi Arabia, research on SE in pediatric patients remains limited, and more research is needed based on local hospital data [3].

SE management follows a three-tiered approach: intravenous (IV) benzodiazepines (BDZs) as the first-line therapy, IV anti-seizure medications (ASMs) for persistent SE, and sedatives such as midazolam for refractory SE (RSE) requiring intensive care unit (ICU) admission [4]. The American Epilepsy Society (AES) guidelines recommend administering BDZs within 5–10 min of seizure onset, ASMs as the second-line therapy within 20–40 min, and general anesthetics as the third-line therapy beyond 60 min [4]. Despite the recommendations, adherence to the guidelines remains inconsistent. A study comparing AES guidelines with protocols from ten U.S. hospitals found that only one hospital adhered to the guidelines, while others implemented more aggressive approaches [5].

Delays in administration can worsen patient outcomes; each minute from seizure onset to the emergency department (ED) increases the risk of the SE lasting >60 min by 5% [6]. Moreover, a study of 218 patients showed that delayed BDZ administration (>10 min) increases the odds of death (adjusted odds ratio (AOR) = 11.0), prolonged seizures (AOR = 2.6), hypotension (AOR = 2.3), and the need for infusion (AOR = 1.8) [7]. In addition, a study of 33,814 cases reported a 1.8% 30-day mortality, 4.6% 1-year mortality, and a 10.7% neurological disability rate [8]. Notably, the time to first BDZ administration has been positively correlated with the time to seizure cessation, as well as the progression to second- and third-line treatments and occurrence of respiratory complications [9].

The inappropriate dosing of BDZs is another concern. A multicenter study enrolled 293 children with RSE and found that 36% received more than two doses of BDZs before escalating to second-line ASMs [10]. Another study investigated 162 episodes of SE and found that 50.4% of patients had subtherapeutic levels of ASMs, 34.6% required intubation, and 1.2% died. Nevertheless, new-onset of seizures are more common in younger children than in those with known epilepsy [11]. Another study showed that only 29.8% of 102 patients received guideline-recommended dosing [12]. In addition, a multicenter study of 542 episodes of SE reported that SE cessation was achieved in 42% of episodes after first-line therapy and in 35% after second-line therapy, whereas 22% of episodes required rapid sequence intubation [13].

The efficacy of ASMs as a second-line therapy varies. One study reported that levetiracetam controls seizures in 60% of pediatric SE cases, compared to 50% with phenytoin, with fewer side effects [14]. Similarly, the Established Status Epilepticus Treatment Trial (ESETT) reported seizure cessation in 47% of episodes using levetiracetam, 45% using fosphenytoin, and 46% using valproate [15].

Moreover, determining the principal cause of SE is challenging because of diverse etiologies [16]. A study in Jeddah identified febrile seizures (30.5%) as the leading cause of SE [17]. Another study conducted in Riyadh found that most cases lack documentation of the specific causes of SE episodes [18]. Due to limited data on this topic in Saudi Arabia, this study evaluated SE management in pediatric patients admitted to the ED of a large tertiary hospital in Riyadh, Saudi Arabia. This study investigated patient characteristics, precipitating factors, responses to different medications, dosing appropriateness, and treatment failures, to support evidence-based therapy for SE in Saudi Arabia.

## 2. Methods

### 2.1. Patient Enrollment and SE Definition

This retrospective observational study evaluated the outcomes of pediatric patients (≤18 years old) with SE who were admitted to the ED of a large teaching hospital in Riyadh, Saudi Arabia. Data were collected from patients with SE admitted between January 2015 and December 2023. SE was defined as two or more seizures without a return to consciousness between seizures or a continuous seizure episode lasting at least five minutes. RSE was defined as continuous seizure episodes that persisted even after the administration of BDZs and one adequately dosed second-line ASM [19]. Patients presenting to the ED meeting the definition for SE or RSE were eligible for inclusion. Conversely, individuals whose seizures were effectively managed or stabilized in the pre-hospital setting or prior to ED arrival were excluded from the study cohort. Ethical approval was obtained from the Institutional Review Board (IRB) of King Saud University Medical City (E-21-5886), with the need for informed consent being waived due to the retrospective nature of the study.

### 2.2. Data Collection

Data were retrospectively collected from electronic medical records of patients and included multiple variables and demographic data, such as age, sex, weight in relation to the date of the event, history of epilepsy, and other comorbidities. Home medications (ASMs) for patients with epilepsy were also documented. The study outlined probable causes, as documented in the patients’ files, such as infections, hypoglycemia, poor sleep hygiene, trauma, and changes in ASMs. Data on the treatments provided for SE episodes in the ED included agents, dosages, routes of administration, and clinical responses to each administered agent. The length of hospital stay and the outcomes of the visit, whether discharged, admitted to the wards or ICU, or death during hospitalization, were recorded. Patients with incomplete medical records were excluded.

### 2.3. Study Outcomes and Evaluation

This study examined pediatric patients with SE admitted to the ED in Saudi Arabia and possible precipitating factors for SE episodes. The main outcome of the study was the medication effectiveness in each medication round, which was defined as the termination of seizures within 20 min of the start of therapy, no seizure recurrence within 60 min, and no need for additional therapy [4]. The institution has adopted standardized clinical practice guidelines for the management of SE, integrating its diagnostic criteria and therapeutic protocols with the consensus definitions outlined by the AES and the National Institute for Health and Care Excellence (NICE) [4,20]. The study further analyzed the association between the response to treatment in the first medication round and patient characteristics to identify predictors of treatment failure in our pediatric population. Furthermore, for each medication round of BDZs or ASMs, records of different ASMs and BDZs were administered and patient responses were collected to analyze their effectiveness in terminating SEs among pediatric patients in each medication round.

All three BDZs (diazepam, lorazepam, and midazolam), along with phenytoin and levetiracetam, were stored in designated locations within the ED to ensure immediate availability when required. Prior to 2019, BDZs were housed in restricted-access cabinets, while phenytoin and levetiracetam were stored separately in dedicated compartments. Following system upgrades, all aforementioned medications became readily accessible through automated dispensing units within the ED.

A BDZ/ASM medication round was considered effective if the last dose of the drug was administered before the clinical resolution of the SE episode, with no SE recurrence during the rest of the hospital stay. This classification enabled an accurate evaluation of the therapeutic efficacy and provided insights into the pharmacological management of SE in pediatric patients. The following doses were used as references to assess the appropriateness of dosing in pediatric patients with SE based on the AES guideline: diazepam (0.15–0.2 mg/kg), lorazepam (0.1 mg/kg), midazolam (10 mg for patients > 40 kg; 5 mg for patients 13–40 kg.), fosphenytoin (20 mg/kg), levetiracetam (60 mg/kg), phenobarbital (15–20 mg/kg), phenytoin (15–20 mg/kg), valproate (40 mg/kg), pentobarbital (5–15 mg/kg), and propofol (1–2 mg/kg) [4].

### 2.4. Statistical Analysis

Descriptive statistics, mean ± standard deviation (SD) for continuous data, and frequencies with percentages (%) for categorical data were used to summarize demographic and clinical data. The *t*-test and chi-square test for continuous and categorical variables, respectively, were used to assess differences between responders and non-responders as well as study outcomes based on the patients’ baseline characteristics. All *p*-values < 0.05 were considered statistically significant. All data were analyzed using SAS software (version 9.4; SAS Institute Inc., Cary, NC, USA).

## 3. Results

### 3.1. Demographic Characteristics, Etiologies for SE Episodes, and Home Medications

The study included 487 episodes of SE among pediatric patients, with a mean age of 6.1 ± 4.1 years and a mean weight of 20.8 ± 13.0 kg. Most patients were male (279/487, 57.3%) with a documented history of epilepsy (361/487, 74.1%). The etiology of the current SE episode was unknown in most patients (306/487; 62.8%), while the most documented etiology or trigger was infection (77/487; 15.8%). Regarding home medications, most patients were taking levetiracetam (248/487, 50.9%). While 74.1% of these patients had epilepsy documented in their medical records, 30.8% (150/487) had no history of treatment with ASMs. The details are summarized in Table 1.

### 3.2. Medication Rounds and Responses to BDZs and ASMs

Of the 487 SE episodes, BDZs were used in the first medication round to treat 404 episodes (83.0%), whereas the remaining 83 episodes (17.0%) were treated using ASMs in the first medication round without preceding BDZ treatment. Only 44 of the 404 SE episodes (10.9%) resolved with BDZs, whereas 66.3% of episodes (55/83) resolved in patients who received ASMs. The overall response or resolution of SE episodes to any medication in one medication round ranged from 20.3% in the first medication round to 58.3% in the fifth medication round. Overall, BDZs were used on the last medication round that lead to patients’ response in 10.9–54.6% of episodes. Nevertheless, ASMs were used on the last medication round that lead to patients’ response in 33.3–77.1% of episodes (Table 2).

**Figure 1:**
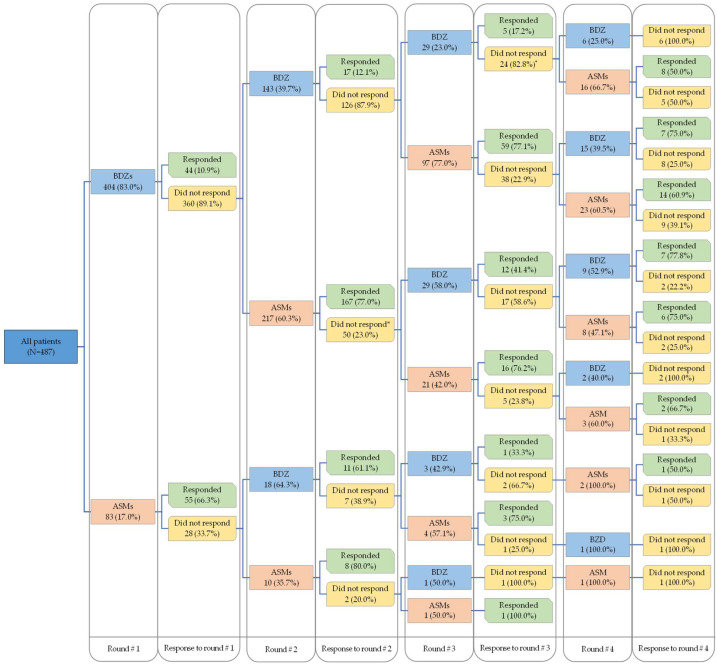
The sequence of pharmacological intervention provided and patients’ responses. * The remaining received infusion.

In the second medication round, 17.4% (28/161) of the episodes resolved with BDZs while 77.1% (175/227) of the episodes resolved with ASMs, regardless of whether the same drug had been administered in the first medication round. When considering the medication administered in the first medication round, only 12.1% (17/143) of the episodes that were treated with two consecutive doses of BDZs responded in the second medication round, while 80.0% (8/10) of the episodes responded when patients received two consecutive doses of ASMs (Table 2 and Figure 1).

In terms of specific medications, lorazepam was the most frequently administered drug in the first medication round when BDZs were chosen as the initial therapy (360/404; 89.1%), and only 10.3% (37/360) of the SE episodes were resolved. Notably, the pediatric response to diazepam in the first medication round was better (5/20; 25%). Among the episodes in which patients received ASMs in the first medication round, levetiracetam was the most used drug (58/83; 69.9%), and 75.9% (44/58) of the episodes resolved when levetiracetam was chosen as the starting drug. In subsequent medication rounds, similar patterns of use and response to BDZs or ASMs were observed, with equal or slightly better responses to medications. These findings are presented in Table 2.

### 3.3. Outcomes Based on Patient Characteristics and Treatment in the First Medication Round

Overall, 191/487 episodes (39.3%) stabilized, and patients were discharged from the ED. The remaining 296/487 episodes (60.7%) were admitted to the neurological ward (152/296, 51.4%) or ICU (144/296, 48.6%). Patients admitted to hospitals were significantly younger than those who were discharged (5.5 ± 4.4 vs. 7.1 ± 4.0 years; *p* < 0.0001), with an equal distribution between males and females. Patients with a history of epilepsy were more likely to be discharged from the ED than patients without a history of epilepsy (46.3% vs. 19.0%; *p* < 0.0001). The hospitalization rate was higher for patients who received BDZs than for those who received ASMs in the first medication round (63.9% vs. 45.8%; *p* = 0.0021). Most patients were appropriately dosed in the first medication round (385/487; 79.1%), and the appropriateness of medication dosing in the first medication round was not associated with the outcome for these patients. Further details regarding the study outcomes are provided in Table 3.

### 3.4. Patient Responses to Medications in Each Medication Round

A total of 1318 medication rounds were used to manage the 487 SE episodes. Of these medication rounds, 33.5% of patients in appropriately dosed medication rounds responded (315/940; 33.5%), whereas underdosed medication rounds had more favorable responses (177/378; 46.8%). Regarding the appropriateness of dosing and responses to each medication group, most patients who received BDZs were appropriately dosed (619/794; 77.9%). Their overall response rate was 14.5% (90/619), whereas underdosed patients (175/794; 22.1%) had a slightly better response (36/175; 20.5%). For the medication rounds of ASMs, 60.8% (313/515) were appropriately dosed and had a much better response than rounds of BDZs (217/313; 69.3%). A similar response (140/202, 69.3%) was observed in the underdosed ASM rounds (202/515, 39.2%). Lorazepam was the most frequently used BDZ (546/794; 68.8%) and was appropriately administered in 86.1% of the rounds; however, the response rate was 12.8% after appropriate dosing and 7.8% after underdosing. Levetiracetam was the most frequently used ASM, with a response rate of 75.5% after appropriate dosing, compared to 77.7% in underdosed rounds (Table 4).

### 3.5. Factors Associated with Patient Responses in the First Medication Round

While age, sex, and a history of epilepsy were not associated with the probability of responding to the first round of medication, the effectiveness of the first medication round was significantly affected by the type of medication and dosing accuracy. Patients who received ASMs had a more favorable response than those who received BDZs (66.3% vs. 10.9%; *p* < 0.0001). However, underdosed medications were associated with a better response in the first medication round than appropriately dosed medications (28.4% vs. 18.2%; *p* = 0.0222). Detailed results are presented in Table 5.

## 4. Discussion

This study aimed to investigate and evaluate the current management of SE episodes in pediatric patients admitted to the ED and to identify triggering factors and assess their response to different treatments. Despite the global prevalence of SE in children, research on its management in Saudi Arabia is limited, particularly regarding adherence to treatment guidelines and the effects of dosing accuracy on outcomes. Notably, the initial administration of ASMs was associated with a significantly better resolution of SE than that of BDZs, underscoring the critical importance of treatment selection and dosing accuracy in each population, to optimize patient outcomes.

Our study identified distinct demographic patterns of SE episodes in pediatric patients, with 57.3% of cases occurring in males and 42.7% in females. The average age of the patients was 6.1 ± 4.1 years. This demographic distribution is consistent with a study conducted in Mogadishu, Somalia, which reported a mean age for pediatric patients with SE of 6 ± 4.7 years and a male-to-female ratio of 54.3% to 45.7% [1]. A similar age distribution was observed in a study conducted in Portugal, and a similar sex distribution was observed in a study conducted in Iowa, USA [2,11]. Among the cases in our study, most were attributed to unknown etiologies (62.8%), which aligns with a previous study in Riyadh that attributed most episodes to unknown reasons [18]. This contrasts with the findings of a study conducted in Jeddah, Saudi Arabia, which primarily attributed SE to febrile causes [17]. Moreover, most episodes in our study occurred in patients with underlying epilepsy (74.1%), which aligns with a study from Iowa, USA, in which 69% of episodes occurred in patients previously diagnosed with epilepsy [11]. These discrepancies may be attributed to differences in population characteristics or access to healthcare services.

Levetiracetam was the most commonly used home medication among our patients, accounting for 50.2% of prescriptions. This finding is similar to that of a prospective cohort study in Nottingham, UK, in which levetiracetam was prescribed to 50% (62/124) of pediatric epilepsy cases. Another study conducted in Gujarat, India found that levetiracetam was the most common monotherapy prescribed to pediatric patients [21]. In contrast, a study conducted in Jordan reported that valproic acid was the most frequently used home medication (50%) [22]. These differences may reflect regional variations in prescription practices, treatment guidelines, or the availability of medications in different countries at the time of the study.

Our study showed that 79.1% (385/487) of pediatric patients received appropriately dosed medications in their first medication round, resulting in a direct ED discharge rate of 39.0%, which was similar to the rate in underdosed patients (102/487; 20.9%), of which 40.2% were discharged from the ED without admission. Thus, the proportion of hospitalized patients was comparable between patients that received the appropriate dose and those that were underdosed (61.0% vs. 59.8%). This finding suggests that the appropriate dosing in our study did not significantly influence the outcomes of pediatric patients in the ED, which may be attributable to variations in disease severity, pharmacokinetic profiles, or undocumented delays in medication administration.

Age plays a significant role in the outcome of SE episodes. Patients admitted to the medical wards or ICU were significantly younger than those discharged from the ED (5.0 ± 4.0 vs. 7.1 ± 4.0 years; *p* < 0.0001). However, sex did not significantly influence the outcomes of SE episodes. Furthermore, patients admitted to the ICU had the longest hospital stays, averaging 21.1 ± 87.6 days, compared to those discharged from the ED (0.4 ± 0.3 days) and admitted to medical wards (6.3 ± 9.7 days). This aligns with a study conducted in Iowa, USA, which found that children with new-onset SE were younger (median [IQR] age: 3 [1,2,3,4,5,6,7,8] years), than children with a known seizure disorder (median age: 4 years); however, the sex, race, insurance status, seizure type, intubation requirements, and mortality were not significantly different [11].

Similarly, sex did not significantly influence SE outcomes in our study population. Likewise, Fetta et al. found no statistically significant difference in hospitalization rates between male and female patients (71.4% vs. 82%; *p* = 0.287) [23]. In contrast, findings from the ESETT pediatric subgroup reported much shorter hospital and ICU stays (median: 3 and 2 median days, respectively) for levetiracetam- and fosphenytoin-treated children [15]. This difference can be explained by population characteristics, treatment protocols, and healthcare system capacity.

Most patients with a pre-existing diagnosis of epilepsy in our cohort required hospital admission. Our results contrast with those from a study conducted in Iowa, USA, in which the lengths of hospital stay were not different between patients with or without a history of epilepsy [11]. This may indicate that most SE episodes in our study were due to poor management of epilepsy, although this was not documented as the main etiology of the SE episode. This calls for better management of epilepsy in pediatric patients, especially in young patients who require hospitalization or ICU admission. Furthermore, the reasons for this poor outcome among younger patients require further evaluation to identify possible methods to improve epilepsy management in the youth.

Furthermore, our study evaluated the response to medication based on the appropriateness of the medication dosing in pediatric patients in the first medication round and across all medication rounds. Although most pediatric patients were appropriately dosed in most medication rounds (940/1318 medication rounds; 71.3%) of either medication group, the response rate was lower in appropriately dosed rounds of therapy (33.5%) than for underdosed rounds (46.8%). Specifically, a total of 378 instances of subtherapeutic dosing were identified in our study, comprising 175 rounds of dosing involving BDZs and 202 involving ASMs. Although the response rate for underdosed BDZs was notably low at 20.5%, it was even lower with appropriately dosed rounds of BDZs (14.5%). In comparison, underdosed ASMs demonstrated a response rate of 69.3%, which was consistent with the 69.3% response rate for appropriately dosed ASMs. In a retrospective cohort study, only 31% of pediatric patients with SE received adequate benzodiazepine dosing (≥4 mg lorazepam-equivalent). Although 75% of those patients who developed RSE received inadequate dosing, neurological outcomes and mortality were not significantly affected [24]. Therefore, this poor response to appropriate dosing can be explained by the overall poor response to BDZs or possibly due to the delay in BDZ administration, which was not documented in our study (90/619 medication rounds, 14.5%), not just due to the underdosing of these medications.

The better responses in the underdosed medication rounds can be explained by the acceptable response to ASMs, regardless of the appropriateness of the administered doses (69.3% for appropriately dosed and underdosed medication rounds); however, BDZs were more frequently used than ASMs in our study. This unexpected outcome suggests that factors beyond dosing accuracy, such as the timing of administration, patient-specific characteristics, severity of SE episodes, or even patient pharmacokinetics, pharmacodynamics, or pharmacogenetics, may further influence the effectiveness of these medications in pediatric patients with SE or epilepsy. Moreover, providers in practice sometime administer a fixed lower dose of levetiracetam instead of the weight-based dosing for management of SE. In a small retrospective cohort study, seizures ceased after a 1 g fixed dose of levetiracetam in approximately 31% of cases; however, the difference was not statistically significant from the rate seen in the ESETT trial [25]. However, it is worth noting that this was a small-scale study and further evidence of this practice is still needed, and there is ongoing research to investigate the effectiveness of this practice [26].

As previously indicated, patient responses to BDZs were generally low. Although lorazepam was the most commonly administered BDZ, it had the lowest response rate in appropriately dosed patients (12.8%). Midazolam (bolus) was more effective when underdosed than when appropriately dosed (30.6% vs. 15.6%). Diazepam showed inconsistent results, achieving a 32.5% positive response when appropriately dosed, but no response when underdosed. These variations highlight the complexity and variations in the effectiveness of BDZ, warranting further research on these medications in our population.

The suboptimal response to BDZs in SE may, in part, be attributed to the uncertain duration of seizure activity prior to ED presentation. Experimental and clinical evidence suggests that the therapeutic efficacy of BDZs diminishes over time, potentially due to mechanisms such as GABA-A receptor internalization and upregulation of NMDA receptor activity [27,28]. Nevertheless, additional clinical observations indicate that the attenuation of pharmacological responsiveness correlates with the prolonged duration or recurrence of SE itself, irrespective of the timely administration of multiple BDZ doses [6].

In contrast, ASMs demonstrated a significantly better overall response rate than BDZs, with similar effectiveness between appropriately dosed and underdosed medication rounds (69.3%). Levetiracetam was the most effective ASM, showing a positive response in >75% of cases, regardless of the dose administered. The better response to ASMs may be influenced by the large number of patients who received levetiracetam and had a positive response, which was better than the responses following administration of other ASMs. Phenytoin rounds showed better responses to underdosing than to appropriate dosing (72.4% vs. 61.6%). Valproate exhibited 100% efficacy, regardless of the dosing accuracy; however, it was used in a very small number of patients (six patients), which may limit the generalizability of this finding. These results suggest that most ASMs maintain their effectiveness even when underdosed, likely because of their broad therapeutic index and favorable pharmacokinetic profiles. When comparing these findings to those of a study conducted in India, a notable difference was observed in the response rates to ASMs. In our study, the overall response to ASMs was 66.3%, whereas the Indian study showed higher responses of 78% for levetiracetam, 88% for phenytoin, and 92% for valproate [21]. However, our results agree with the findings of the ESETT, which also reported nearly equal efficacies of levetiracetam (52%), fosphenytoin (49%), and valproate (52%), irrespective of the dosing precision [15,29]. ASMs, including levetiracetam, were more effective than BDZs in our population, warranting early switching to ASMs in patients with refractory SE. These results support the development of personalized treatment regimens to optimize SE management in young patients.

The effects of demographic characteristics, history of epilepsy, and type of medication used were investigated to determine the factors associated with patient responses in the first medication round. In general, the patient responses in the first medication round were not related to age, sex, or history of epilepsy. However, the choice of medication significantly affected the response rates in the first medication round. ASMs were notably more effective than BDZs, with response rates of 66.3% and 10.9%, respectively (*p* < 0.0001). Moreover, the dosing accuracy also influenced treatment responses, and patients who were underdosed had a more favorable response than those receiving appropriate doses, regardless of the medication used (28.4% vs. 18.2%; *p* = 0.0222). However, this unusual result can be explained by the previous observation that most medication rounds of BDZs in our study were appropriately dosed but ineffective, whereas the medication rounds of ASMs were 69.3% effective, regardless of the appropriateness of the dose.

We suggest conducting a regional study similar to the ESETT to evaluate the effectiveness of different BDZs and ASMs in patients with epilepsy and SE, while considering all previously highlighted factors. The findings of this study will improve the responses of pediatric patients to these medications by providing a better understanding of the pharmacodynamic and pharmacokinetics of these medications in our study population and improving the dosing. Consequently, patient outcomes and the health-related quality of life would improve. Thus, we emphasize individualized dosing strategies for BDZs and ASMs in pediatric patients with epilepsy or SE. Furthermore, dosing protocols can be standardized based on these studies, and the timing of administration can be optimized for better treatment outcomes.

This study provides valuable insights into treatment approaches for pediatric patients with SE in an emergency setting. Nevertheless, certain limitations must be acknowledged. Owing to its retrospective nature, this study relied on the accuracy and completeness of the medical workup data. Thus, inconsistencies or omissions with respect to documentation of the diagnosis could have led to the missed identification of SE cases, especially those not captured through medication records alone. In addition, patients who experienced SE but did not receive BDZs or ASMs during their ED visits may have been unintentionally excluded. This limitation potentially underestimates the true burden of SE and limits the generalizability of our findings to the entire population encountered in emergency settings. Moreover, pre-hospital care data could not be included in the study because the emergency medical service records were not merged with hospital documentation. Therefore, SE episodes that were managed entirely in the field or at least partially stabilized before arrival at the ED were not assessed, which restricted the study from evaluating the full spectrum of SE care pathways in Saudi Arabia. The starting times of SE episodes were not documented in patients’ files; therefore, we were unable to use this data to evaluate the effect of delayed therapy on treatment response. Additionally, the actual etiologies of most SE episodes were not recorded, which hindered further investigation into the relationship between different etiologies and patient outcomes. We thus recommend that such data be systematically collected in the future to enable researchers to better assess the impact of delayed therapy and various etiologies on patient outcomes. Despite these limitations, this study had several strengths. The study comprised nearly a decade of patient data in one of the largest retrospective cohorts of pediatric SE in a Saudi tertiary care setting. Detailed information on actual treatment patterns regarding the types of medications, accuracy of dosages, and response rates were provided. The study contributed to local data because of the focus on regional practices and highlighted major gaps in adherence to international guidelines. Furthermore, the assessment of treatment efficacy over multiple medication rounds provided an understanding of escalation strategies and response variability.

## 5. Conclusions

This study reveals critical gaps in pediatric management and real-world SE settings in Saudi Arabia. ASMs, particularly levetiracetam, demonstrate superior effectiveness compared to BZDs, regardless of the dosing accuracy, indicating that existing first-line therapies for SE may need re-evaluation based on local efficacy and effectiveness studies. Relevant findings highlight the vital aspects of the timely and efficient prevention of complications for better outcomes. Establishing national protocols tailored to local practices and conducting prospective multicenter studies are crucial for improving the quality of care and standardizing treatments for pediatric SE in the region.

## Figures and Tables

**Table 1:** Demographics, possible etiologies for the SE episodes, and home medications.

Characteristics	Overall (*n* = 487)
**Age, years (mean ± SD)**	6.1 ± 4.1
**Weight, kg**	20.8 ± 13.0
**Body mass index, kg/m^2^**	16.5 ± 3.9
**Gender**	
Male	279 (57.3)
Female	208 (42.7)
**History of epilepsy**	361 (74.1)
Documented etiologies/triggers *	
Infection	77 (15.8)
Non-compliance to ASMs	42 (8.6)
Trauma	13 (2.7)
Hypoglycemia	10 (2.1)
Poor sleep hygiene	9 (1.8)
Change in ASMs	6 (1.2)
Unknown reason	306 (62.8)
Home medication(s) *	
Levetiracetam	248 (50.9)
Carbamazepine	85 (17.5)
Topiramate	65 (13.3)
Valproic acid	62 (12.7)
Phenobarbital	58 (11.9)
Lamotrigine	19 (3.9)
Phenytoin	8 (1.6)
Vigabatrin	8 (1.6)
No previous treatment	150 (30.8)

* Some patients had more than one home medication or more than one possible etiology/trigger while others had no home medication or no documented etiology/triggers. Abbreviations: ASM—anti-seizure medication.

**Table 2:** The number and percentage of medication rounds of treatment and their success in terminating SE episodes on each round of treatment.

Medication	Round # 1 Resolved/Rounds	Round # 2 Resolved/Rounds	Round # 3 Resolved/Rounds	Round # 4 Resolved/Rounds	Round # 5 Resolved/Rounds	Round # 6 Resolved/Rounds
**Overall (BDZs/ASMs)**	99/487 (20.3)	203/388 (52.3)	97/185 (52.4)	42/88 (47.7)	28/48 (58.3)	12/23 (52.2)
**BDZs**	44/404 (10.9)	28/161 (17.4)	18/62 (29.0)	9/33 (27.3)	10/23 (43.5)	6/11 (54.6)
Lorazepam	37/360 (10.3)	17/124 (13.7)	8/36 (22.2)	2/17 (11.8)	0/7 (0.0)	2/2 (100.0)
Midazolam (bolus)	2/24 (8.3)	9/24 (37.5)	10/23 (43.5)	4/12 (33.3)	10/15 (66.7)	4/9 (44.4)
Diazepam	5/20 (25.0)	2/13 (15.4)	0/3 (0.0)	3/4 (75.0)	0/1 (0.0)	---
**ASMs**	55/83 (66.3)	175/227 (77.1)	79/123 (64.2)	31/53 (58.5)	14/21 (66.7)	3/9 (33.3)
Levetiracetam	44/58 (75.9)	117/146 (80.1)	50/64 (78.1)	10/18 (55.6)	8/12 (66.7)	2/4 (50.0)
Phenobarbital	1/7 (14.3)	11/20 (55.0)	9/19 (47.4)	1/6 (16.7)	3/4 (75.0)	0/3 (0.0)
Phenytoin	9/17 (52.9)	43/57 (75.4)	20/40 (50.5)	19/28 (67.9)	3/5 (60.0)	1/2 (50.0)
Valproate	1/1 (100.0)	4/4 (100.0)	---	1/1 (100.0)	---	---
Sedation *	---	---	---	2/2 (100.0)	4/4 (100.0)	3/3 (100.0)

* The sedation was induced using midazolam infusion. The numbers in the table are number of SE cases resolved/number of medication rounds (%). Abbreviations: BDZ—benzodiazepines; ASM—anti-seizure medication.

**Table 3:** Outcomes of care based on patients’ characteristics and treatment used on first round of therapy.

Characteristics	Discharged from ED	Admitted to Hospital			*p*-Value *
		Overall	Ward	ICU	
**Overall**	191/487 (39.3)	296/487 (60.7)	152/296 (51.4)	144/296 (48.6)	
**Age**	7.1 ± 4.0	5.5 ± 4.4	5.9 ± 4.1	5.0 ± 4.0	**<0.0001**
**Gender**					0.9367
Male	109/279 (39.1)	170/279 (60.9)	86/170 (50.6)	84/170 (49.4)	
Female	82/208 (39.4)	126/208 (60.6)	66/126 (52.4)	60/144 (47.6)	
**History of epilepsy**					**<0.0001**
No	24/126 (19.0)	102/126 (81.0)	45/102 (44.1)	57/102 (55.9)	
Yes	167/361 (46.3)	194/361 (53.7)	107/194 (55.2)	87/194 (44.8)	
**Medication used first**					**0.0021**
BDZs (*n* = 404)	146/404 (36.1)	258/404 (63.9)	129/258 (50.0)	129/258 (50.0)	
ASMs (*n* = 83)	45/83 (54.2)	38/83 (45.8)	23/38 (60.5)	15/38 (39.5)	
**Dosing of medication**					0.8203
Appropriate (*n* = 385)	150/385 (39.0)	235/385 (61.0)	126/235 (53.6)	109/235 (46.4)	
underdosed (*n* = 102)	41/102 (40.2)	61/102 (59.8)	26/61 (42.6)	35/61 (57.4)	
Length of stay, days **	0.4 ± 0.3	13.5 ± 61.9	6.3 ± 9.7	21.1 ± 87.6	**0.0003**

Results are presented as mean ± SD or frequency (%). * The *p*-values are from the comparison between patients who were discharged from the ED and patients who were admitted to the hospital at any level of care. ** Two patients were excluded from the length of stay analysis as they have died after long hospitalization. Abbreviations: BDZ—benzodiazepines; ASM—anti-seizure medication.

**Table 4:** Overall response to medications based on appropriateness of dosing on all rounds of therapy.

Rounds of Therapy	Did Not Respond	Responded
**Overall rounds (BDZ/ASMs)**		
Appropriate dosing	625/940 (66.5)	315/940 (33.5)
Underdosed	201/378 (53.2)	177/378 (46.8)
**BDZ**		
Appropriate dosing	529/619 (85.5)	90/619 (14.5)
Underdosed	139/175 (79.4)	36/175 (20.6)
**Lorazepam**		
Appropriate dosing	410/470 (87.2)	60/470 (12.8)
Underdosed	70/76 (92.1)	6/76 (7.9)
**Midazolam (bolus)**		
Appropriate dosing	92/109 (84.4)	17/109 (15.6)
Underdosed	68/98 (69.4)	30/98 (30.6)
**Diazepam**		
Appropriate dosing	27/40 (67.5)	13/40 (32.5)
Underdosed	1/1 (100.0)	0/1 (0.0)
**ASMs**		
Appropriate dosing	96/313 (30.7)	217/313 (69.3)
Underdosed	62/202 (30.7)	140/202 (69.3)
**Levetiracetam**		
Appropriate dosing	43/176 (24.4)	133/176 (75.6)
Underdosed	28/126 (22.2)	98/126 (77.8)
**Phenytoin**		
Appropriate dosing	46/120 (38.3)	74/120 (61.7)
Underdosed	8/29 (27.6)	21/29 (72.4)
**Phenobarbital**		
Appropriate dosing	7/16 (43.8)	9/16 (56.2)
Underdosed	26/42 (61.9)	16/42 (38.1)
** Valproate**		
Appropriate dosing	0/1 (0.0)	1/1 (100.0)
Underdosed	0/5 (0.0)	5/5 (100.0)
Sedation *		
Appropriate dosing	0/8 (0.0)	8/8 (100.0)
Underdosed	0/1 (0.0)	1/1 (100.0)

Results are presented as frequency (%). * This group only includes one medication: midazolam (infusion). Abbreviations: BDZ—benzodiazepines; ASM—anti-seizure medication.

**Table 5:** Response to benzodiazepines or anti-seizure medications on first medication round based on demographic characteristics and past medical history.

Variable		Response on First Medication Round		*p*-Value *
	Categories	Did Not Respond	Responded	
**Age, years (mean ± SD)**		6.1 ± 4.1	6.3 ± 4.1	0.5933
**Gender**	Male	216 (77.4)	63 (22.6)	0.1526
	Female	172 (82.7)	36 (17.3)	
**History of epilepsy**	No	106 (84.1)	20 (15.9)	0.1489
	Yes	282 (78.1)	79 (21.9)	
**Medication used**	BDZ	360 (89.1)	44 (10.9)	**<0.0001**
	ASM	28 (33.7)	55 (66.3)	
**Dosing of medication**	Appropriate	315 (81.8)	70 (18.2)	**0.0222**
	Underdosing	73 (71.6)	29 (28.4)	

Results are presented as mean ± SD or frequency (%). Abbreviations: BDZ—benzodiazepines; ASM—anti-seizure medication. * The *p*-values are from the chi-square test for categorical data or *t*-test for continuous data.

## Data Availability

The data that support the findings of this study are available on request from the corresponding author. The data are not publicly available due to privacy or ethical restrictions.

## Abbreviations

The following abbreviations are used in this manuscript:

SE	Status Epilepticus
RSE	Refractory Status Epilepticus
ED	Emergency Department
BDZs	Benzodiazepines
ASMs	Anti-Seizure Medications
AES	American Epilepsy Society
IV	Intravenous
ICU	Intensive Care Unit
AOR	Adjusted Odds Ratio
ESETT	Established Status Epilepticus Treatment Trial
IRB	Institutional Review Board
SD	Standard Deviation
